# A rare case of liver regenerative and non-neoplastic lesion resembling a well-differentiated hepatocellular carcinoma

**DOI:** 10.1186/s40792-024-01820-1

**Published:** 2024-02-01

**Authors:** Kosuke Hirose, Takeo Toshima, Taro Tobo, Satohiro Kai, Masakazu Hirakawa, Satoshi Higuchi, Takashi Ofuchi, Kiyotaka Hosoda, Yusuke Yonemura, Yuichi Hisamatsu, Takaaki Masuda, Shinichi Aishima, Koshi Mimori

**Affiliations:** 1 Department of Surgery, Kyushu University Beppu Hospital, 4546, Shoen, Beppu-Shi, Oita-Ken 874-0838 Japan; 2 Department of Pathology, Kyushu University Beppu Hospital, 4546, Shoen, Beppu-Shi, Oita-Ken 874-0838 Japan; 3 Department of Radiology, Kyushu University Beppu Hospital, 4546, Shoen, Beppu-Shi, Oita-Ken 874-0838 Japan; 4 Department of Pathology and Microbiology, Saga University, Nabeshima 5-1-1, Saga, 849-8501 Japan

**Keywords:** Regenerative lesion, Nodular regenerative hyperplasia, NRH-like lesion, Laparoscopic partial resection, Fatty liver

## Abstract

**Background:**

Nodular regenerative hyperplasia (NRH) is a rare disease that presents pathologically as diffuse hepatic nodules without fibrous septa. It is believed to be caused by vasculopathy against a background of various systemic diseases, such as hematologic, autoimmune, and drug-induced diseases, with various symptoms. In spite of the recent imaging advances, various atypical cases of nodular lesions are observed in daily clinical practice. Cases that do not completely meet these criteria are referred to as -like or -similar lesions in clinical situations, making it difficult to understand their pathogenesis. We present a case in which two hepatic nodular lesions were noted and difficult to differentiate from malignancy preoperatively. The lesions were laparoscopically resected and a pathological diagnosis with non-neoplastic liver regenerative nodules resembling NRH was made.

**Case presentation:**

A 49-year-old man with no alcohol or drug intake and no past medical history was identified as having liver tumors on screening examination without any symptoms. Contrast-enhanced computed tomography (CT) showed two hepatic tumors; approximately 2-cm tumors at S7 and S8. Gadolinium-ethoxybenzyl-diethylenetriamine-pentaacetic acid (Gd-EOB-DTPA)-enhanced magnetic resonance imaging (MRI) revealed fat inclusions in their contents. Ethoxybenzyl (EOB) uptake was also observed during the hepatobiliary phase. Based on preoperative examinations, we suspected well-differentiated hepatocellular carcinoma (HCC) and performed laparoscopic S7/8 partial resection for these lesions. Macroscopically, the resected specimens showed a non-cirrhotic yellowish-cut surface containing brownish, ill-defined lesions with irregular borders. Microscopically, these lesions showed zonal necrosis, congestion, and aggregation of hemosiderin-laden macrophages around the central vein. In these areas, the fatty deposition of hepatocytes was lower than that in the surrounding background hepatocytes. Histopathologically, neither neoplastic nor hyperplastic lesions were observed, and he was diagnosed as regenerative hepatic change with centrilobular necrosis.

**Conclusions:**

Considering the pathological results, these lesions were thought to be a type of NRH-like lesion with possible hepatic vessel disorder. However, the lesion’s cause and classification was difficult to determine. The accumulation of these regenerative changes accompanying fatty liver is needed to clarify the mechanism and its clinical significance.

## Background

Hepatic nodular regenerative hyperplasia (NRH) is a rare pathological disorder in which normal liver parenchymal cells are diffusely replaced with nodules without fibrous septa. It is often considered asymptomatic unless complicated by portal hypertension and subsequent varices, ascites, or splenomegaly [1–6], and NRH was found in only 2.6% in a large autopsy analysis of 2500 cases [7]. On the other hand, NRH could be more common in chemotherapy-related liver injury, as NRH was identified in 87 of 406 (18.2%) patients with colorectal liver metastases resected after chemotherapy [8]. Grossly, the liver is replaced by diffuse nodules of 1–3 mm in size, and there is generally no fibrosis between the nodules unlike in cirrhosis. Pathologically, the hepatocytes become small and atrophic between the nodules; by contrast, the sinusoids become dilated. The final diagnosis is made pathologically, considering the history of the disease. On liver needle biopsy, these regenerative and atrophic changes are often difficult to detect just with conventional hematoxylin and eosin staining, and special stains, such as reticulin staining, should be considered [9].

Hepatocellular atrophic areas between nodules are likely to be associated with obstructive changes in the portal vein and are thought to be the result of multiple nodule formation as a compensatory response to decreased and altered blood flow [10].

The prevalence of asymptomatic focal liver lesions (FLLs) detected using imaging modalities, such as computed tomography (CT) and magnetic resonance imaging (MRI), has increased owing to the wider use of such modalities in clinical practice [11]. Focal liver lesions were defined as solid or cystic masses identified as abnormal areas in the liver. The diagnostic approach and management of these lesions have been summarized in the guidelines of the Practice Parameters Committee and the Board of the Trustees of the American College of Gastroenterology [12]. Examining lesions that may be malignant is primarily based on three-phase contrast CT and contrast MRI. Almost all hepatocellular carcinoma (HCC) cases > 2 cm are currently diagnosed solely using imaging examinations [13].

Liver diseases showing nodular lesions on imaging include focal nodular hyperplasia, partial nodular transformation, and idiopathic portal hypertension, other than NRH. The diagnosis and classification of these hepatic nodular lesions are generally based on a combination of the presence of neoplastic lesions, number, size, and location of nodules, background liver disease complications, imaging, and pathology [14]. Among these nodular lesions, various atypical cases are observed in daily clinical practice. These lesions sometimes include cases that do not completely meet the diagnostic criteria and are referred to as -like lesions. They are difficult to understand in terms of pathogenesis.

In this report, we describe a case of pathologically regenerative and non-neoplastic changes in the liver with no significant history other than mild fatty liver, which was diagnosed after laparoscopic partial hepatectomy. Two nodular lesions closely resembling well-differentiated HCC were observed on preoperative imaging.

## Case presentation

**Fig. 1:**
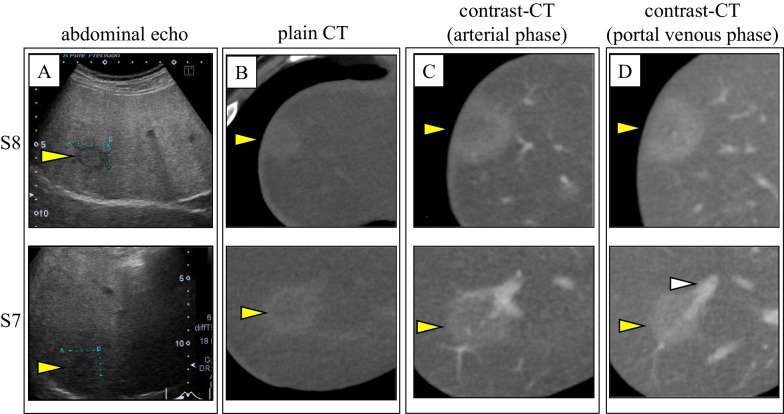
Ultra-sonographic (**A**), plain (**B**), and contrast computed tomography (CT) (**C** arterial phase; **D** portal venous phase) of the lesions. The upper images refer to the S8 lesion, and the images below refer to the S7 lesion. The yellow arrowheads indicate the lesions, and the white one indicates portal branch for S7

**Fig. 2:**
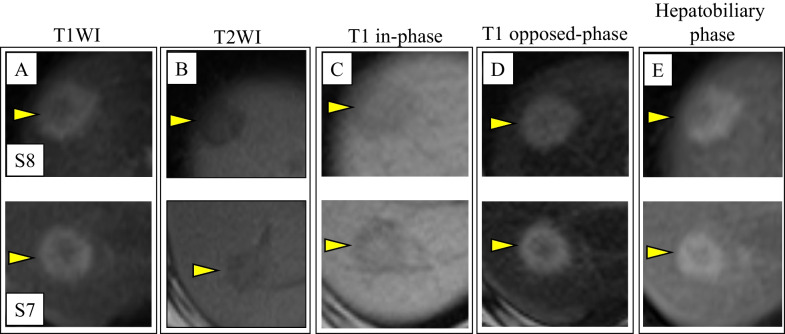
Various findings of magnetic resonance imaging (MRI) of the lesions: S8 (upper) and S7 (below) lesions by T1- and T2-weighted images (T1WI: **A** and T2WI: **B**), T1-weighted in- (**C**) and opposed-phase (**D**), and ethoxybenzyl-contrast images in hepatobiliary phase (**E**) are shown. The yellow arrowheads indicate the each lesion

A 49-year-old man with no specific medical or familial history was diagnosed with two liver tumors on regular abdominal sonography. He had no alcohol, medication, or health supplement intake, or a history of hepatic virus infection. Physical examination showed no jaundice, edema, gynecomastia, or palmar erythema, and his body mass index was 22.7. Blood examination showed no specific findings, including normal alpha-fetoprotein and protein induced by vitamin K absence-II levels and no preoperative physiologic disorder. Abdominal sonography revealed two hepatic tumors at S8: 2.1 cm and S7: 2.2 cm. They also showed clear hypoechoic lesions with mildly fatty liver (Fig. 1A). Both of them showed iso- to high-density areas in plain CT (Fig. 1B). Contrast-enhanced CT revealed delayed contrast (Fig. 1C, arterial phase; Fig. 1D, portal venous phase of the lesions). MRI images revealed that the tumors showed high-intensity at their borders and low-intensity at their contents on T1-weighted images (Fig. 2A). The tumors showed low-intensity on T2-weighted images (Fig. 2B). The images also revealed that the central areas of the both tumors showed high-intensity in T1 in-phase (Fig. 2C) and low-intensity in T1 opposed-phase of MRI (Fig. 2D), which meant that the tumors included fat components. The images also showed Ethoxybenzyl (EOB) uptake of the lesions in the hepatobiliary phase with Gadolinium-ethoxybenzyl-diethylenetriamine-pentaacetic acid (Gd-EOB-DTPA)-enhanced MRI (Fig. 2E). Based on these findings, we suspected well-differentiated HCC based on fatty liver, or benign liver nodules like NRH or focal nodular hyperplasia (FNH)-like lesions as differential diagnosis.

**Fig. 3:**
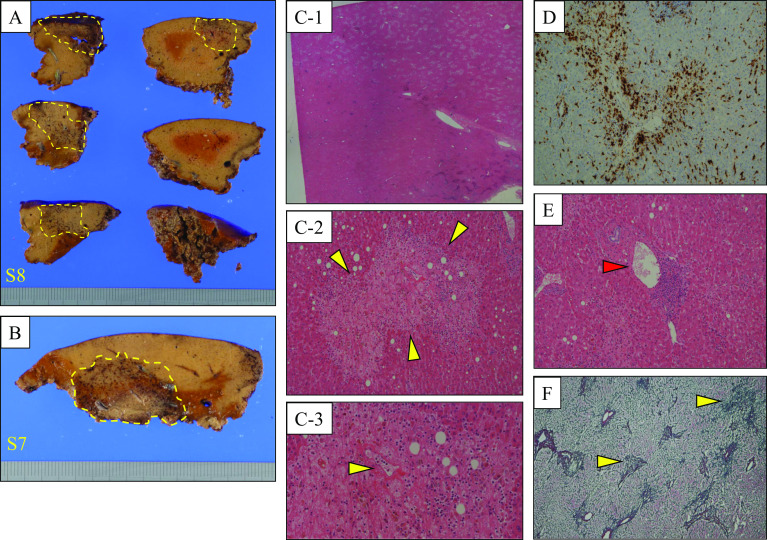
Macroscopic and microscopic findings of the resected specimen: macroscopic images of the resected specimens of S8 (**A**) and S7 (**B**). Microscopic images of the resected lesion with hematoxylin and eosin (HE) staining are shown in **C** (**C-1**, loupe view; **C-2**, magnified by ×100; **C-3**, magnified by ×200). The ill-defined lesion is pointed by some yellow arrowheads (**C-2**). Central venous area of the lesion is magnified by ×400 (**C-3**). The yellow arrowhead indicates the central vein, and loss of hepatocyte and congestion is observed (**C-3**). Accumulated macrophages around the central vein are revealed by CD68 stain (**D**). Non-specific mild lymphocytic infiltrate is observed at the periportal region with HE staining (**E**). The red arrowhead indicates the portal vein. The yellow arrowheads indicate the dense reticular fibers with atrophic hepatocytes surrounding the central veins by silver staining at the lesion (**F**)

Laparoscopic S7/8 partial resection was performed for both treatment and diagnosis with his consent. During surgery, the liver appeared fatty, and there was no tumor exposure at the surface level. Sonography revealed hypoechoic and round tumors with regular borders at S7 and S8. Each tumor included the hepatic vein and Glisson’s capsules. The total bleeding volume was 120 mL with an operative time of 205 min. The patient was discharged 10 days after the operation without any complications. Macroscopically, the resected specimens showed signs of a mild fatty liver with a yellowish background with ill-defined brownish lesions and irregular borders (Figs. 3A: S8 and B: S7). Microscopically, approximately 30% of the background hepatocytes showed macrovesicular steatosis with hematoxylin and eosin (HE) staining (Fig. 3C-1, loupe view; Fig. 3C-2, magnified by ×100; Fig. 3C-3, magnified by × 200). Pathologically, the indefinite nodular lesion (2 cm in size) showed zonal hepatocyte loss associated with congestion and hemosiderin-laden macrophages around the central vein (Fig. 3C-2 and C-3). One of ill-defined lesion is pointed by some yellow arrowheads in Fig. 3C-2, which include degenerated hepatocyte around central vein, congestion, and hepatocellular area with poor fat deposition. No fibrosis or septum formation was observed around the nodules. Macrophage accumulation in the centrilobular area was highlighted by CD68 staining (Fig. 3D). No hepatocyte atrophy or macrophage accumulation was observed in other background livers. Non-specific lymphocytic infiltrates were observed in the portal area (Fig. 3E). Atrophic hepatocytes and dense reticular fibers around the central veins were clearly distinguishable by silver staining (Fig. 3F). There were no tumor lesions, including hyperplasia, adenoma, or malignancies, and no obvious microvascular changes, such as thrombus formation. Based on those pathological findings, we diagnosed regenerative hepatic change with centrilobular necrosis.

## Discussion

We report a case of benign regenerative nodular lesion of the liver that closely resembled well-differentiated HCC on imaging and was difficult to diagnose preoperatively. Furthermore, the pathological investigation of the resected specimen revealed hepatocyte desquamation and macrophage accumulation around the central vein, consistent with the nodal area, with lipid deposition in the background liver. None of fibrous septum around the nodules, obvious vessel obstruction, and tumorous components was observed. There was no history of underlying diseases, drug use, or special preferences, and the patient was considered to have NRH-like lesions with a history of fatty liver disease.

First, this case involved a nodular lesion whose malignancy could not be ruled out on preoperative imaging, leading to a diagnosis of regenerative nodular lesion after laparoscopic resection. Owing to the unique nature of blood flow control in the liver, various benign nodular lesions occur because of abnormal blood flow, and various atypical cases are occasionally observed and reported in clinical practice [5, 6]. The differentiation between malignant and benign tumors, including HCC, is clinically important, and the gold standard diagnostic modality is contrast-CT and enhanced-MRI. With the development in imaging technologies, the majority of HCC larger than 2 cm can be differentiated using imaging alone [4]. In the present case, both liver lesions had a maximum diameter of approximately 2 cm. However, the possibility of highly differentiated HCC could not be ruled out on preoperative imaging, and resection was performed partly for the purpose of accurate diagnosis. As shown by Luo et al. [15], preoperative diagnosis of benign liver tumors, including NRH, that lack clinical symptoms relies heavily on imaging studies, and diagnosis of HCC leading to resection sometimes occurs. They reported that the preoperative accuracy of CT for the group including NRH was 0% (0/11 cases), and that of MRI was 12.5% (1/8 cases). They also reported that the rare benign liver tumors were most likely to be misdiagnosed as HCC (CT: 16/25 cases, MRI: 16/24 cases), and most of the cases with the unclear diagnosis were difficult to distinguish from HCC (CT: 12/27 cases, MRI: 10/19 cases), especially from well-differentiated HCC, as in this case.

Second, this case is unique because of the characteristic pathology of the resected specimen. A background liver with hepatocytes and approximately 30% fatty deposits was observed, and the lesion showed hepatocyte desquamation, stasis, and phagocytic macrophage accumulation around the central vein specifically, while a non-specific lymphocytic infiltrate was observed in the portal vein area. There were no neoplastic lesions, such as hyperplasia or carcinoma, fibrosis of the septal wall, or multiple small lesions. The distribution of lesions suggested necrosis due to stasis of hepatocytes around the central vein from obstruction of the outflow tract of the hepatic vein tract. However, no stasis symptoms or laboratory findings, such as heart failure or fluid retention, were observed, and microvascular thrombi in the hepatic veins were not identified on pathological and preoperative CT/MRI images. The micro-congestive findings like this without systemic congestion might suggest a kind of atypical NRH-like lesion. The possibility of drug-induced liver injury remained; however, no history of specific medications, including dietary supplements and imported foods, was confirmed. Furthermore, Croquelois et al. showed that inducible inactivation of Notch1 in mice could promote hepatocyte proliferation and lead to the development of NRH without vascular lesions [16], and it suggest that NRH could occur with proliferative signal without microvascular disorder. Therefore, we suspected that the hepatic regenerative changes meant NRH-like lesion.

The patient differs from a typical NRH such that (1) there are only two obvious lesions and no multiple lesions around the liver, (2) there is no pathological evidence of hyperplasia, and (3) there is no background suggesting typical systematic diseases that could be related to NRH, such as rheumatoid arthritis or drug-related factors [8, 17, 18]. Furthermore, atypical cases of noncirrhotic benign nodular lesions are included in LRN by International Working Party [19], and then, atypical NRH-like lesion such as the present case could be called LRN in a broader sense. In this case, preoperative biopsy might have been effective, but was not an option because of the risk of spreading the lesion and possibility that biopsy might not be negative in a well-differentiated HCC due to the lower tumor volume. In this regard, laparoscopic partial resection, which could reduce the bleeding, complications, and abdominal wall destruction [20] with safety [21], is useful. Although there was an option to continue imaging follow-up, the patient was willing to proceed with the diagnosis. After discussing the treatment plan, we decided to opt for surgery.

In conclusion, we present an unusual case of regenerative hepatic nodular lesion that was not easily diagnosed preoperatively. Reports on non-neoplastic nodular localized hepatic lesions are scarce, and further accumulation of these cases is necessary to understand their pathogenesis and improve preoperative diagnostic capabilities.

## Data Availability

Data sharing is not applicable to this article, as no datasets were generated or analyzed in the current study.

## Abbreviations

NRH: Nodular regenerative hyperplasia
CT: Computed tomography
Gd-EOB-DTPA: Gadolinium-ethoxybenzyl-diethylenetriamine-pentaacetic acid
MRI: Magnetic resonance imaging
EOB: Ethoxybenzyl
HCC: Hepatocellular carcinoma
FLL: Focal liver lesion
FNH: Focal nodular hyperplasia
LRN: Large regenerative nodule
HE: Hematoxylin and eosin

## References

[1] Graf L, Dobrota R, Jordan S, Wildi LM, Distler O, Maurer B (2018). Nodular regenerative hyperplasia of the liver: a rare vascular complication in systemic sclerosis. J Rheumatol.

[2] Wanless IR, Peterson P, Das A, Boitnott JK, Moore GW, Bernier V (1990). Hepatic vascular disease and portal hypertension in polycythemia vera and agnogenic myeloid metaplasia: A clinicopathological study of 145 patients examined at autopsy. Hepatology.

[3] Austin A, Campbell E, Lane P, Elias E (2004). Nodular regenerative hyperplasia of the liver and coeliac disease: potential role of IgA anticardiolipin antibody. Gut.

[4] Breen DP, Marinaki AM, Arenas M, Hayes PC (2005). Pharmacogenetic association with adverse drug reactions to azathioprine immunosuppressive therapy following liver transplantation. Liver Transpl.

[5] Al-Mukhaizeem KA, Rosenberg A, Sherker AH (2004). Nodular regenerative hyperplasia of the liver: an under-recognized cause of portal hypertension in hematological disorders. Am J Hematol.

[6] Daniel F, Cadranel JF, Seksik P, Cazier A, Duong Van Huyen JPD, Ziol M (2005). Azathioprine induced nodular regenerative hyperplasia in IBD patients. Gastroenterol Clin Biol.

[7] Wanless IR (1990). Micronodular transformation (nodular regenerative hyperplasia) of the liver: a report of 64 cases among 2,500 autopsies and a new classification of benign hepatocellular nodules. Hepatology.

[8] Vigano L, Rubbia-Brandt L, Rosa GD, Majno P, Langella S, Toso C (2015). Nodular regenerative hyperplasia in patients undergoing liver resection for colorectal metastases after chemotherapy: risk factors, preoperative assessment and clinical impact. Ann Surg Oncol.

[9] Reshamwala PA, Kleiner DE, Heller T (2006). Nodular regenerative hyperplasia: not all nodules are created equal. Hepatology.

[10] Wanless IR, Solt LC, Kortan P, Deck JHN, Gardiner GW, Prokipchuk EJ (1981). Nodular regenerative hyperplasia of the liver associated with macroglobulinemia. A clue to the pathogenesis. Am J Med.

[11] Smith-Bindman R, Miglioretti DL, Johnson E, Lee C, Feigelson HS, Flynn M (2012). Use of diagnostic imaging studies and associated radiation exposure for patients enrolled in large integrated health care systems, 1996–2010. JAMA.

[12] Marrero JA, Ahn J, Rajender Reddy K, Americal College of Gastroenterology (2014). ACG clinical guideline: the diagnosis and management of focal liver lesions. Am J Gastroenterol.

[13] Di Martino M, De Filippis G, De Santis A, Geiger D, Del Monte M, Lombardo CV (2013). Hepatocellular carcinoma in cirrhotic patients: prospective comparison of US, CT and MR imaging. Eur Radiol.

[14] Wanless IR (1995). Special article terminology of nodular hepatocellular lesions. Hepatology.

[15] Luo L, Wang T, Cheng M, Ge X, Song S, Zhu G (2023). Rare benign liver tumors that require differentiation from hepatocellular carcinoma: focus on diagnosis and treatment. J Cancer Res Clin Oncol.

[16] Croquelois A, Blindenbacher A, Terracciano L, Wang X, Langer L, Radtke F (2005). Inducible inactivation of Notch1 causes nodular regenerative hyperplasia in mice. Hepatology.

[17] Jain P, Patel S, Simpson HN, Silver RM, Lewin DN, Campbell RC (2021). Nodular regenerative hyperplasia of the liver in rheumatic disease: cases and review of the literature. J Investig Med High Impact Case Rep.

[18] Hartleb M, Gutkowski K, Milkiewicz P (2011). Nodular regenerative hyperplasia: evolving concepts on underdiagnosed cause of portal hypertension. World J Gastroenterol.

[19] International Working Party (1995). Terminology of nodular hepatocellular lesions. Hepatology.

[20] Kaneko H, Otsuka Y, Kubota Y, Wakabayashi G (2017). Evolution and revolution of laparoscopic liver resection in Japan. Ann Gastroenterol Surg.

[21] Ban D, Ogura T, Akahoshi K, Tanabe M (2018). Current topics in the surgical treatments for hepatocellular carcinoma. Ann Gastroenterol Surg.

